# Protein–RNA specificity by high-throughput principal component analysis of NMR spectra

**DOI:** 10.1093/nar/gku1372

**Published:** 2015-01-13

**Authors:** Katherine M. Collins, Alain Oregioni, Laura E. Robertson, Geoff Kelly, Andres Ramos

**Affiliations:** 1 Molecular Structure Division, MRC National Institute for Medical Research, London NW7 1AA, UK; 2 Research Department of Structural and Molecular Biology, University College London, London WC1E 6BT, UK; 3 MRC Biomedical NMR Centre, MRC National Institute for Medical Research, London NW7 1AA, UK

## Abstract

Defining the RNA target selectivity of the proteins regulating mRNA metabolism is a key issue in RNA biology. Here we present a novel use of principal component analysis (PCA) to extract the RNA sequence preference of RNA binding proteins. We show that PCA can be used to compare the changes in the nuclear magnetic resonance (NMR) spectrum of a protein upon binding a set of quasi-degenerate RNAs and define the nucleobase specificity. We couple this application of PCA to an automated NMR spectra recording and processing protocol and obtain an unbiased and high-throughput NMR method for the analysis of nucleobase preference in protein–RNA interactions. We test the method on the RNA binding domains of three important regulators of RNA metabolism.

## INTRODUCTION

Understanding how RNA-binding proteins select target RNA sequences is key to explain specificity in RNA regulation, but is a challenging problem. Many protein regulators recognize a diverse ensemble of cellular RNA targets by the combined action of several RNA binding domains (1). Recognition of specific RNA sequences by the individual domains is important, and it has been shown that mutations in their RNA target sequences disrupt protein function. However, the sequence specificity of many domains is still debated and the rules of protein–RNA recognition are not fully understood (2).

**Figure 1.**
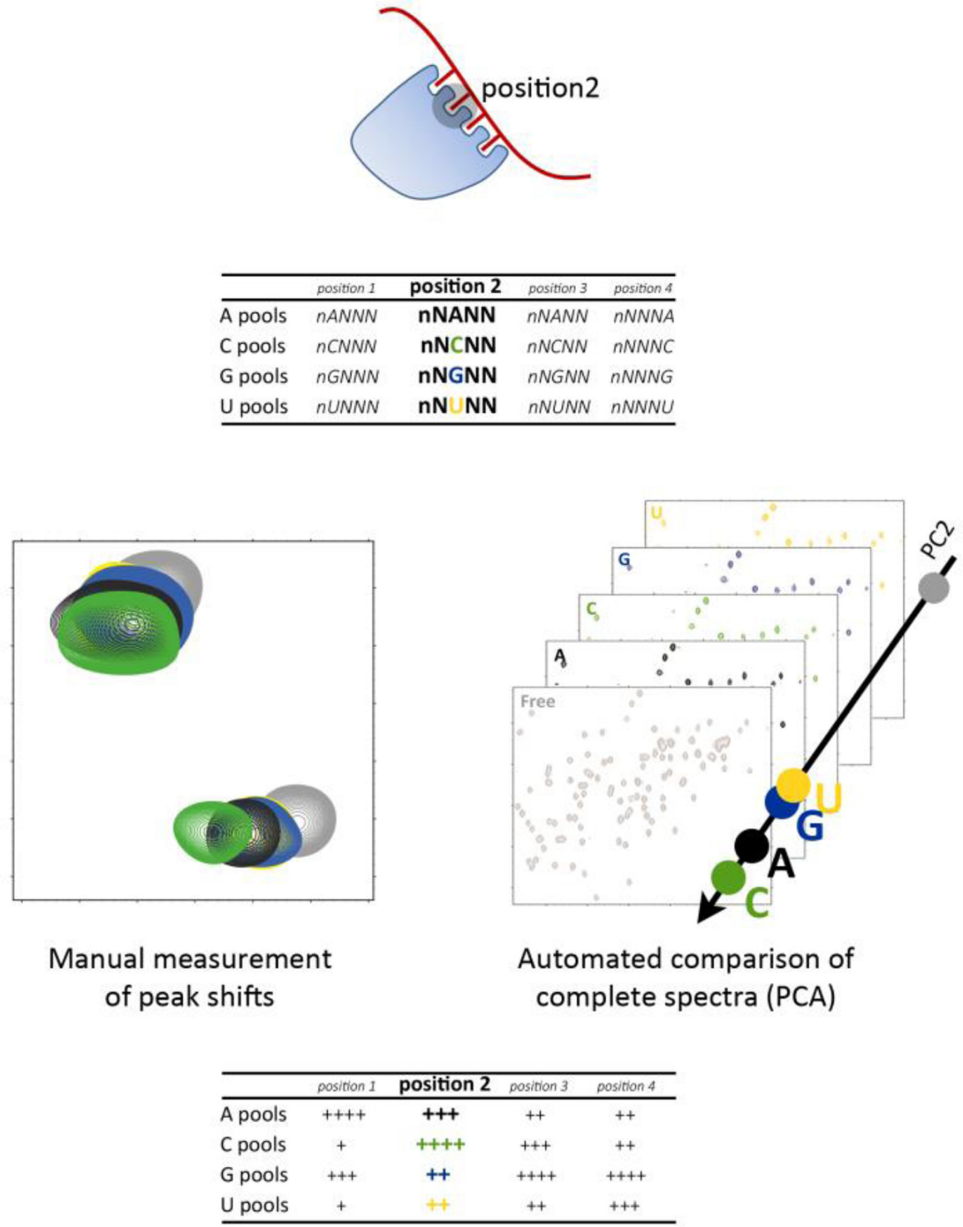
NMR analysis of the sequence preference of an RNA binding domain. To examine the nucleobase preference in position 2, four randomized RNA pools which differ only in the nucleobase in position 2 are added to the protein domain in independent assays (top). The NMR spectra of the four different complexes and of the free protein can be compared either by manually measuring the shift of an ensemble of peaks (by manual analysis—left) or by an automated comparison of the complete spectra using principal component analysis (by PCA—right). The comparison reports on the different average binding affinity of the four pools and allows ranking of the preference of the domain for A, C, G and U in position 2 using comparative scores (bottom).

The scaffold independent analysis (SIA) approach can be used to define the sequence preference of a protein domain. In SIA, ^1^H{^15^N} correlation nuclear magnetic resonance (NMR) spectroscopy is used to compare the average binding affinity of a protein domain to different sets of quasi-degenerate RNA pools (3). For each position in the nucleic acid whose specificity is to be evaluated, four RNA pools are used where this position is occupied by either A, C, G or U, while all other positions are occupied by a randomized mixture of the four bases (Figure 1). The only difference between the four pools is the specified base and therefore the comparison of the average binding affinities of the protein for the four pools reports directly on its nucleobase preference. SIA analysis is performed by first measuring the change in the chemical shift (Δδ) of ∼10 individual protein resonances in four independent titrations with the four individual pools. Then the Δδ of each peak in each titration is normalized with respect to the largest Δδ for that peak observed in any of the four titrations. Lastly, the normalized values across the different peaks in one titration are averaged to obtain the final comparative SIA score (3). These scores reflect the preference of the protein for one nucleobase versus another. We and others have tested the SIA protocol described above on a small number of KH and RRM domains and we have shown that it can provide novel and functionally relevant information on the domains’ sequence preference (3–7). However, manual measurement and tabulation of peak shifts in SIA analysis is laborious, and the choice of the peaks to be evaluated can introduce bias (4).

Here we describe a novel semi-quantitative application of principal component analysis (PCA) to analyse similar NMR data in a high-throughput fashion and determine the sequence preference of an RNA binding domain. PCA was developed as a technique to reduce the complexity of multi-variable sets of data to a smaller number of new variables (the principal components) that capture the majority of the variability existing in the data. It allows for the identification of patterns in the data displaying the data in a way that highlight differences and similarities. In the current work, PCA is employed to recast five variables (free, A, C, G, U), each with 290 × 256 data points (the dimensions of the frequency domain ^1^H{^15^N} correlation spectra), into five principal components with associated scores and loadings. PCA has been used to correlate measured NMR observables with conformational transitions, global structural changes and changes in the protein environment (8,9). Further, it has been used to assess directly variability between NMR spectra. For example in metabolomics, where the principal components of NMR spectra from samples from different patients are used to correlate variations in metabolite levels with disease states (10) and to analyse qualitative changes in ^1^H{^15^N} correlation spectra of proteins resulting from high-throughput ligand screening procedures (11). We have now applied PCA to compare the ^1^H{^15^N} correlation spectra of a protein either free or in the presence of the four individual RNA pools used to assess nucleobase preference in SIA (Figure 1)—and evaluate which pool leads to the largest changes. This is, as far as we are aware, the first time PCA is applied to directly analyse whole NMR spectra in a semi-quantitative rather than qualitative fashion.

Manual recording of NMR spectra during SIA titrations is labour-intensive and requires substantial spectrometer time: in order to complement the unbiased data analysis concept described above we have also re-designed the experimental set up for NMR data acquisition and have established a downstream processing pipeline that converts and processes the NMR data and performs PCA analysis.

The method reported here allows one to compare the specificity of domains in a streamlined fashion and we expect will provide an important tool to characterize RNA target selection by protein regulators. The implementation of the protocol is based on standard NMR equipment and standard software for spectra acquisition and data processing and analysis.

## MATERIALS AND METHODS

### Protein preparation

Samples of the three recombinant proteins were prepared by expressing the constructs in *Escherichia coli*, in minimal media with ^15^NH_4_Cl_2_ sole as nitrogen source and purifying the protein by affinity chromatography and in two cases size-exclusion chromatography. Final experimental conditions were optimized based on the quality of the NMR spectra.

Briefly, Strep-tagged RNA15 RRM domain (residues 2–103) was expressed in BL21(DE3) (Novagen) and purified using Strep-Tactin resin (IBA). The tag was removed with GST-tagged Human Rhinovirus 3C protease overnight. The protease was then removed using a disposable GST column (GE Healthcare). The protein was further purified using size exclusion chromatography on a Superdex 75 (16/60) (GE Healthcare). NMR samples were prepared in 40 mM NaCl, 20 mM Tris pH 7.5, 0.5 mM TCEP.

His-tagged T-STAR KH domain (residues 50–160) was expressed in Rosetta BL21 DE3 and purified using Ni-NTA agarose (Qiagen) followed by TEV cleavage during overnight dialysis in phosphate buffer at 4°C and further purified by size-exclusion chromatography on a Superdex 75 10/300 (GE Healthcare). Final samples were in 50 mM NaCl, 20 mM NaH_2_PO_4_ pH 6.1, 0.1% B-mercaptoethanol.

His-tagged TUT4 CCHC ZnF3 domain (residues 1354–1382) was expressed in BL21(DE3) cells and purified using Ni-NTA agarose followed by TEV cleavage during overnight dialysis in Tris buffer at 4°C. A second reverse Ni-NTA agarose purification removed the His-tag and protease, and lead to high purity samples. Final samples were in 100 mM NaCl, 10 mM Tris pH 7.4, 0.5 mM TCEP, 10 μM ZnCl_2_.

### RNA preparation

All RNAs pools were purchased from Thermo Scientific (Dharmacon). Pools were de-protected following the manufacturer instructions and re-suspended in H_2_O. RNA concentration was determined by UV spectroscopy.

### NMR

Samples of 25 μM RNA15, 40 μM TSTAR, and 100 μM TUT4 were prepared and RNA pools added as required to obtain measurable shifts and depending on the affinity of each protein for RNA and on the overall spectral quality. Protein–RNA ratios were 1:1 for RNA15, 1:2 for TSTAR and 1:4 for TUT4. NMR experiments were performed using 3 mm NMR tubes with a sample volume of 180 μl.

NMR data were acquired using a Bruker Avance III NMR spectrometer operating at 700 MHz and equipped with a 5 mm TCI cryoprobe. Automated sample changing and data acquisition were accomplished using a Samplejet accessory controlled with Topspin 2.1 via IconNMR. Samples were stored at 4°C and then pre-heated at 25°C prior to loading. Locking, tuning, matching and shimming were performed automatically. 2D ^15^N-SOFAST-HMQC experiments with 32 scans, 64 increments and a recycle delay of 250 ms (total experiment time 21 min per sample) gave adequate resolution and sensitivity and were used throughout. For each RNA position, spectra from a control sample and four pools were processed as a pseudo-3D dataset using NMRPipe and passed as input to pcaNMR, a NMRPipe module which employs the NIPALS algorithm to apply PCA to the series of NMR spectra. This efficient algorithm permits the calculation of all five principal components in seconds. An example processing script is available from the authors.

## RESULTS

**Figure 2.**
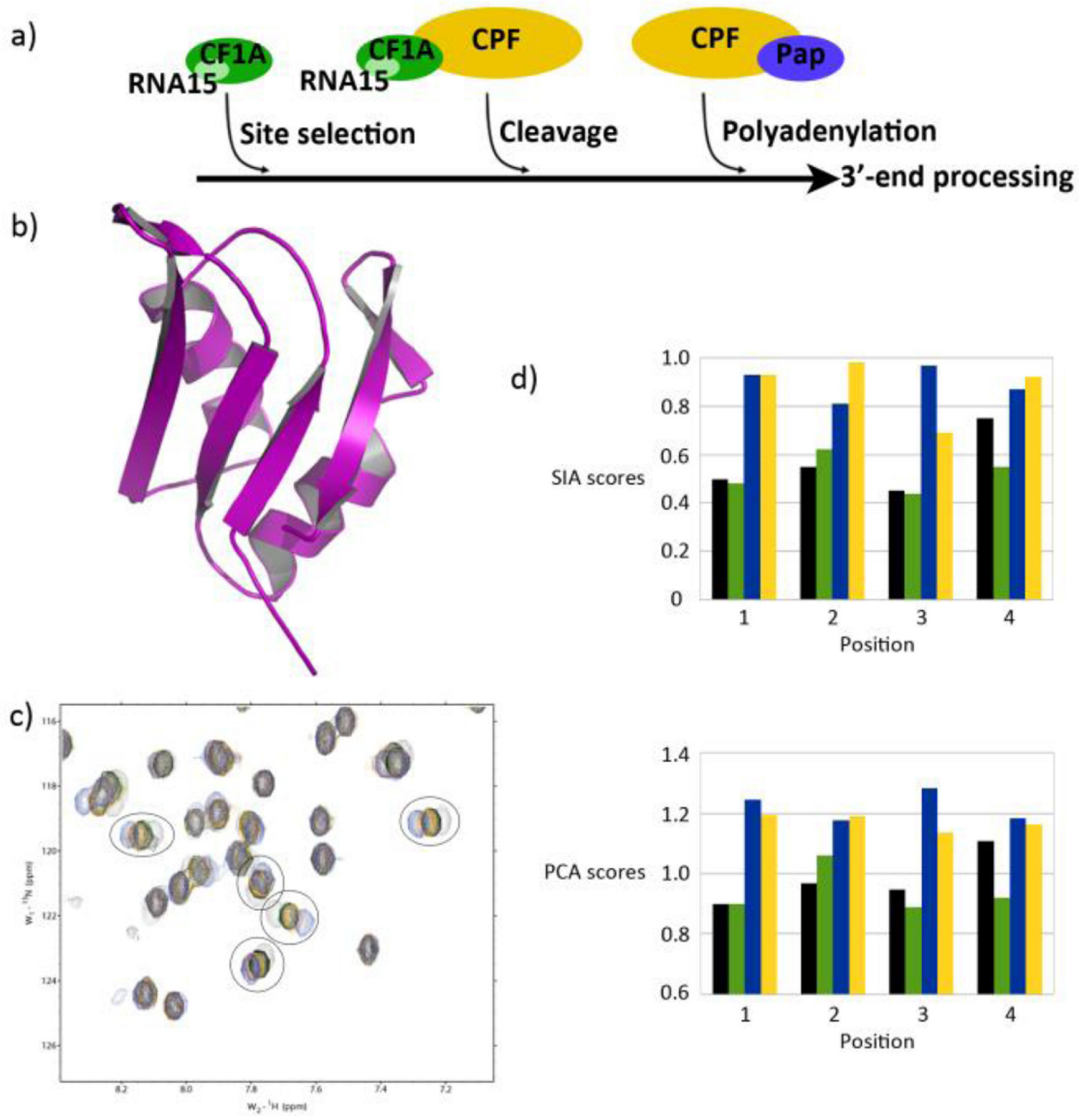
Nucleobase preference of RNA15 RRM domain. (**a**) RNA 15 RRM mediates the docking of the CF1 complex on the nascent RNA chain, which is important for RNA cleavage and polyadenylation. (**b**) Ribbon representation of RNA15 RRM domain (PDB ID: 2X1B). (**c**) Overlay of 2D ^15^N-SOFAST-HMQC spectra from free RNA15 (grey), and RNA15 in complex with each of the four nnnXn pools at protein to RNA ratio 1:1, NNNAN (black), NNNCN (green), NNNGN (blue) and NNNUN (yellow). Five peaks used in the SIA analysis are highlighted and show the order of nucleobase preference of the position. The larger shifts indicate greater affinity for that specific base. These peaks have been manually selected because their chemical shift changes upon RNA binding can be measured accurately. Changes that were difficult to measure accurately because either too small, or because of peak broadening or overlap were not used in the SIA analysis. However, changes of the position and line-width of these resonances contribute to the PCA score. (**d**) Comparison of scores for RNA15 either generated by manual analysis (top) or using PCA (bottom). Histograms display the binding site position on the x-axis with each bar representing the A, C, G or U RNA pools (black, green, blue and yellow respectively). PCA scores are plotted as the difference between the absolute scores of the free spectrum and of the different bound spectra. The absolute values of the reported manual and PCA scores are not directly related as they derive from different evaluation procedure but they are here used in a comparative fashion to assess nucleobase preference.

The method was tested on the RNA Recognition Motif (RRM) domain of the yeast mRNA 3′end processing factor RNA15, a core component of the Cleavage Factor 1A (CF1A) complex (12,13). CF1A, together with the much larger Cleavage and Polyadenylation Factor (CPF) complex performs the 3′ end cleavage and polyadenylation of the pre-mRNA molecule (Figure 2). CF1A is required for both the selection of the cleavage site and for the cleavage itself. It has four core subunits, RNA14, RNA15, PCF11 and Clp1 and interacts both with the Serine-2 phosphorylated C-terminal domain of RNA polymerase II and with short sequence elements in the RNA. The interaction between RNA15 and the 3’UTR of the nascent RNA chain is necessary for CF1A recruitment (14) and is mediated by an RRM domain located in the N-terminal segment of the protein. The structure of the RNA15 RRM, alone and bound to RNA (Figure 2), together with related binding assays have provided an initial assessment of the RNA binding properties of this domain (Figure 2) (15,16). Below we determine the full nucleobase preference of RNA15 RRM, comparing the results obtained by the manual and PCA-based SIA methods.

In the previously described manual SIA approach each of the 16 SIA pools is titrated into the protein sample and spectra are recorded at three different titration points (free protein, plus two different RNA concentrations) (3). In order to streamline NMR data acquisition, we have now recorded a single ^1^H{^15^N}-SOFAST-HMQC spectrum (17) for each of the complexes between the protein and one of the 16 RNA pools plus a single spectra for the free protein. These spectra were acquired using a spectrometer equipped with an automated sample changer and 3 mm NMR tubes. Together, the automatic set up for data acquisition and the new experimental strategy reduces the time of NMR experiments from five days to about nine hours, while using 3 mm tubes halves sample requirement.

After acquisition, the data were converted and processed in-batch using an NMRpipe-based pipeline. We then calculated the principal components for the group of spectra that define nucleobase preference in position 1 using the NMRPipe module pcaNMR (18). The calculation was performed on an ensemble that includes the spectrum of the free protein, which is used as reference to define the basis set for the PCA calculation, plus the four spectra of complexes with NANNN, NCNNN, NGNNN and NUNNN pools. To represent graphically the PCA ranking of the spectra of each of the four protein–RNA complexes with respect to the reference spectrum, we subtracted the absolute value of the loading of a principal component (in this case the second principal component) of the first RNA-bound protein spectrum from that of the reference. The value we obtain represents the distance between the two projections on the second principal component. We repeated this procedure for the first, third, fourth and fifth principal component. The automated pipeline for spectra processing and analysis reduced the time required for the analysis of the data to a few minutes.

An initial analysis of the principal component data revealed that, for all four positions, a clear correlation exists between the loadings on the second principal component and RNA-dependent chemical shift changes, i.e. the spectra where the peaks shift more between free and bound also have a second principal component that is more distant from the reference spectrum—despite this component accounting for only ∼7% of the variance. We did not observe any such correlation for the first, third, fourth and fifth principal components (Supplementary Figure S1). The first principal component (typically ∼80% of the variance) is presumably dominated by changes in peak intensity—the method as used entails a dilution on addition of the RNA pools. The third, fourth and fifth components contribute <5% each of the remaining variance (Supplementary Table S1). This correlation between the second PC and peak shifts has been noted previously in the analysis of qualitative changes in NMR spectra of proteins recorded during high-throughput screening of ligand binding (11).

We then compared the results of manual and PC analysis for the four positions in the RNA target. The absolute values of the SIA scores and of the PCA scores reported in Figure 2 are not directly related as they derive from very different data evaluation procedures. However, both types of scores can be used to compare the differences between the free protein spectrum and the four individual bound protein spectra and to rank the similarities between free and bound spectra. The comparison showed that the ranking of the values of the second principal component recapitulate the ranking of SIA scores obtained by manually measuring the shift of peaks in the same set of spectra. It is worth mentioning that only a subset of peaks with large chemical shift changes is normally used in manual SIA analysis. This can potentially introduce bias, and we have previously reported changes in SIA scores of up to 0.1 when partially overlapping sets of peaks from the same titration are used (4). On the contrary, PCA undertakes a global analysis of the complete spectrum and includes information provided by the whole set of peaks, including the many peaks that undergo small chemical shift changes (Figure 2).

**Figure 3.**
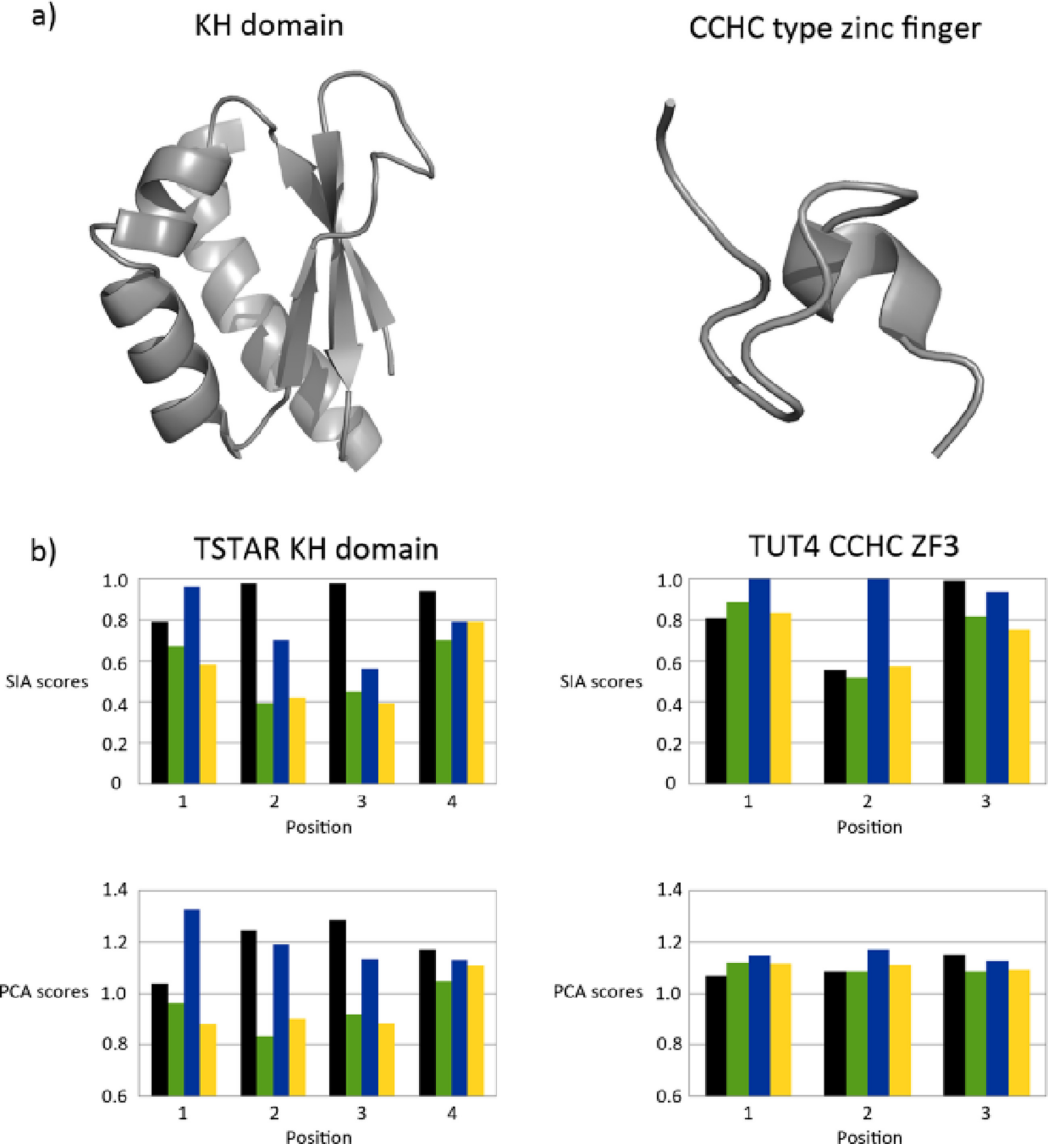
Comparison of nucleobase preference for TSTAR and TUT4 by manual SIA and PCA-based SIA. (**a**) Ribbon representations of example KH (left, KSRP KH3 domain PDB ID: 2HH3) and CCHC zinc finger domains (right, Lin28 ZnF2 domain, PDB ID: 2CQF). (**b**) Comparison of nucleobase preference generated manually (top) or using PCA (bottom). Histograms display the binding site position on the x-axis with each bar representing the A, C, G and U RNA pools (black, green, blue and yellow respectively). PCA scores are plotted as the difference between the absolute scores of the free spectrum and of the different bound spectra.

Next, we extended our analysis and probed the effectiveness of this protocol on two other common RNA binding domains. T-STAR, also referred to as Sam68-like mammalian protein 2 (SLM2) is an important splicing regulator of the *Neurexin* gene (19). The protein contains an extended KH domain (STAR domain), which is thought to bind to A-rich sequences, but whose actual specificity is still to be defined (20). TUT4 is a non-templated poly-uridylase that plays a key role in the biogenesis of the tumour suppressor let-7 miRNA, and that contains three CCHC zinc fingers (21,22). The specificity of these zinc fingers is yet to be characterized, however zinc fingers from viral and mammalian proteins have been shown to bind G/A-rich sequences (23–25). The KH core domain of T-STAR, and the third CCHC ZnF domain of TUT4 (Figure 3) were employed to probe how PCA and SIA approaches compare in performing the analysis of the nucleobase preference of KH and ZnF domains. The protocol used was the same described for the RNA15 RRM domain, except that only three positions were scanned for TUT4 ZnF—based both on the structural information obtained on a CCHC ZnF domain in complex with RNA (24) and on our binding assays on TUT4 ZnF (Collins *et al*., unpublished). Our analysis again showed a comparable ranking of nucleobase preference in PCA and manual approaches both for the KH and ZnF domain.

## DISCUSSION

We present here a principal component-based approach to compare changes in NMR spectra upon binding of RNA oligonucleotides, used as an alternative to manual SIA analysis to determine the specificity of an RNA binding domain. Coupling PCA to automated sample handling, spectrometer control and processing reduces the time required for data acquisition, processing and analysis by an order of magnitude, from several days to several hours.

The use of PCA for the analysis of NMR data is well established. However, the use of PCA as a tool to obtain a semi-quantitative ranking of affinities from the direct analysis of 2D dataset has not been attempted before. A first important question was which, if any, of the principal components in our set of spectra correlates with the shift of resonances in the same NMR spectra. The plots of the first five principal components against the weighted chemical shift average of the peaks used in the manual SIA analysis (Supplementary Figure S1) showed that a good correlation exists only for the second principal component. Such a correlation between chemical shift changes and the second principal component is consistent with the reported use of the second principal component to screen the binding of small molecular weight compounds to a protein, and we observe it consistently for all positions and in all three domains we have examined (11). The second key question was whether the same nucleobase preference would be obtained using manual SIA and PCA. The results show that the nucleobase ranking by PCA recapitulates the one obtained by SIA and is consistent with the known preference of RNA15 RRM for G/U sequences.

Our aim is to implement an analysis strategy that does not require a domain-dependent optimization of the parameters of PC analysis and testing the PCA method on three common RNA binding domains highlights possible difference in the output deriving from the domains different sizes and RNA binding modes. The ZnF domain is significantly smaller than the KH and the RRM domains. The total number of amide resonances in the spectrum of the TUT4 ZnF4 is about one-third of the number of amide resonances in RNA15 RRM, and the number of resonances that shift upon RNA binding is proportionally lower (Supplementary Figure S2). We report that, although the PCA results recapitulate the manual SIA ones for both domains, the range of the PCA scores is significantly smaller for the ZnF domain than for the KH and RRM ones. Indeed examination of the amount of variance accounted for by each PC suggests that the variance is more evenly distributed over the different components (Supplementary Table S1). This suggests that the empirical relationship between spectral features and principal components has been compromised. This observation may serve as a guide to the range of applicability of the PCA-based analysis. In general, it seems that changes in spectra where only very few peaks change position upon RNA binding are more accurately monitored using manual SIA scoring than PCA analysis.

New *in cell* methods (e.g. CLIP and its variants) have identified the ensemble of RNA targets for many different multi-domain regulators of RNA metabolism and localization. However, in many of these proteins multiple domains contribute to recognition and an understanding of target selection requires the full characterization of the sequence preference of each individual domain. The manual SIA method provides the required characterization of nucleobase preference but is laborious and time consuming and not designed for the characterization of a large number of domains. Above we show that PCA and automated NMR can be used to perform an unbiased and high-throughput analysis of RNA-binding data and extends the reach of our analysis to characterize the specificity in a large number of protein–ssRNA interactions. Further, we believe the quantitative use of PCA in directly evaluating protein-ligand binding affinities we present here has the potential to be applied in a broad range of comparative NMR binding assays.

## SUPPLEMENTARY DATA

Supplementary Data are available at NAR Online.

SUPPLEMENTARY DATA
