# Supplementary material for: Protein–RNA specificity by high-throughput principal component analysis of NMR spectra
Source: Nucleic Acids Res. 2015 Jan 13;43(6):e41. doi: 10.1093/nar/gku1372 (PMC4381048; doi:10.1093/nar/gku1372)
Supplement: SUPPLEMENTARY DATA [file supp_43_6_e41__index.html]

Protein–RNA specificity by high-throughput principal component analysis of NMR spectra — SUPPLEMENTARY DATA 

# Protein–RNA specificity by high-throughput principal component analysis of NMR spectra

## SUPPLEMENTARY DATA

**Files in this Data Supplement:**

- SUPPLEMENTARY DATA
