# Supplementary material for: Protein–RNA specificity by high-throughput principal component analysis of NMR spectra
Source: Nucleic Acids Res. 2015 Jan 13;43(6):e41. doi: 10.1093/nar/gku1372 (PMC4381048; doi:10.1093/nar/gku1372)
Supplement: SUPPLEMENTARY DATA [file supp_gku1372_nar-03326-met-f-2014-File003.pdf]

Supplementary Material

Figure S1 - Correlation of principal component scores with chemical shift perturbation. Each plot shows the correlation of free RNA15 RRM (grey), and RNA15 RRM upon addition of NNNAN (black), NNNCN (green), NNNGN (blue), NNNUN (yellow) at protein to RNA ratios of 1:1. The score for the free spectrum is marked with a grey line across the plot for ease of comparison. The x-axis shows the weighted average chemical shift of peaks used in manual analysis. The y-axis shows the calculated score for the (A) first, (B) second, (C) third, (D) fourth, and (E) fifth principal components.

Figure S2 – The comparison of spectral changes upon addition of an RNA pool for RNA15 RRM, T-STAR KH and TUT4 CCHC-ZnF3 shows that because of the smaller size of the ZnF domain a lower number of resonances shift upon RNA binding. Overlaid ^{1}H-^{15}N SOFAST-HMQC spectra of A) free 25µM RNA15 RRM (red) and with NNNGN at protein to RNA ratio 1:1 (blue), B) free 40µM T-STAR KH (red) and with NNNAN at protein to RNA ratio 1:2, C) free 100µM TUT4 CCHC-ZnF3 (red) and with NNGN at protein to RNA ratio 1:4

Table S1 – Percentage variance accounted for by each of the five PCs for each position analysed in RNA15 RRM, TSTAR KH and TUT4 CCHC-ZF3.

FigureS1

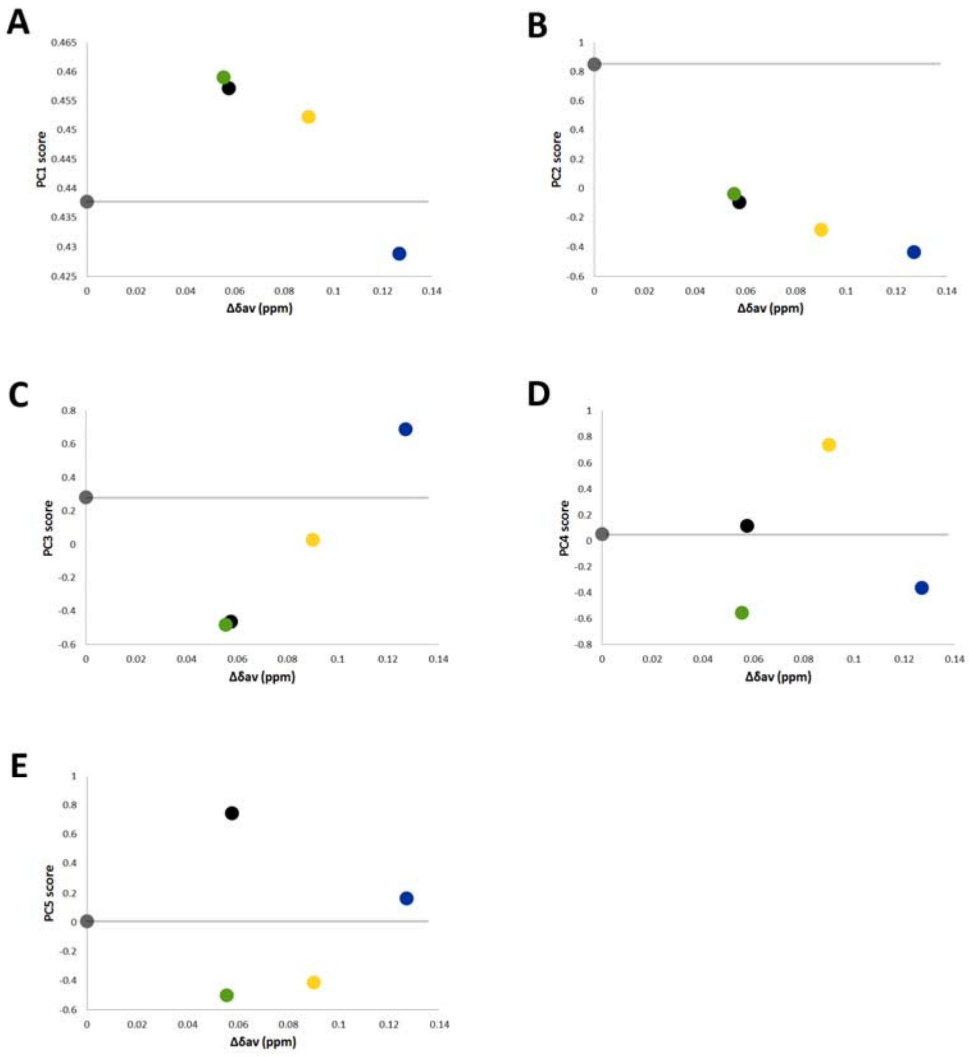
A
0.465
0.46
0.455
0.45
0.445
0.44
0.435
0.43
0.425
PC1 score
0
0.02
0.04
0.06
0.08
0.1
0.12
0.14
Δδav (ppm)
B
1
0.8
0.6
0.4
0.2
0
-0.2
-0.4
-0.6
PC2 score
Δδav (ppm)
C
0.8
0.6
0.4
0.2
0
-0.2
-0.4
-0.6
PC3 score
Δδav (ppm)
D
1
0.8
0.6
0.4
0.2
0
-0.2
-0.4
-0.6
-0.8
PC4 score
Δδav (ppm)
E
1
0.8
0.6
0.4
0.2
0
-0.2
-0.4
-0.6
PC5 score
Δδav (ppm)

FigureS2

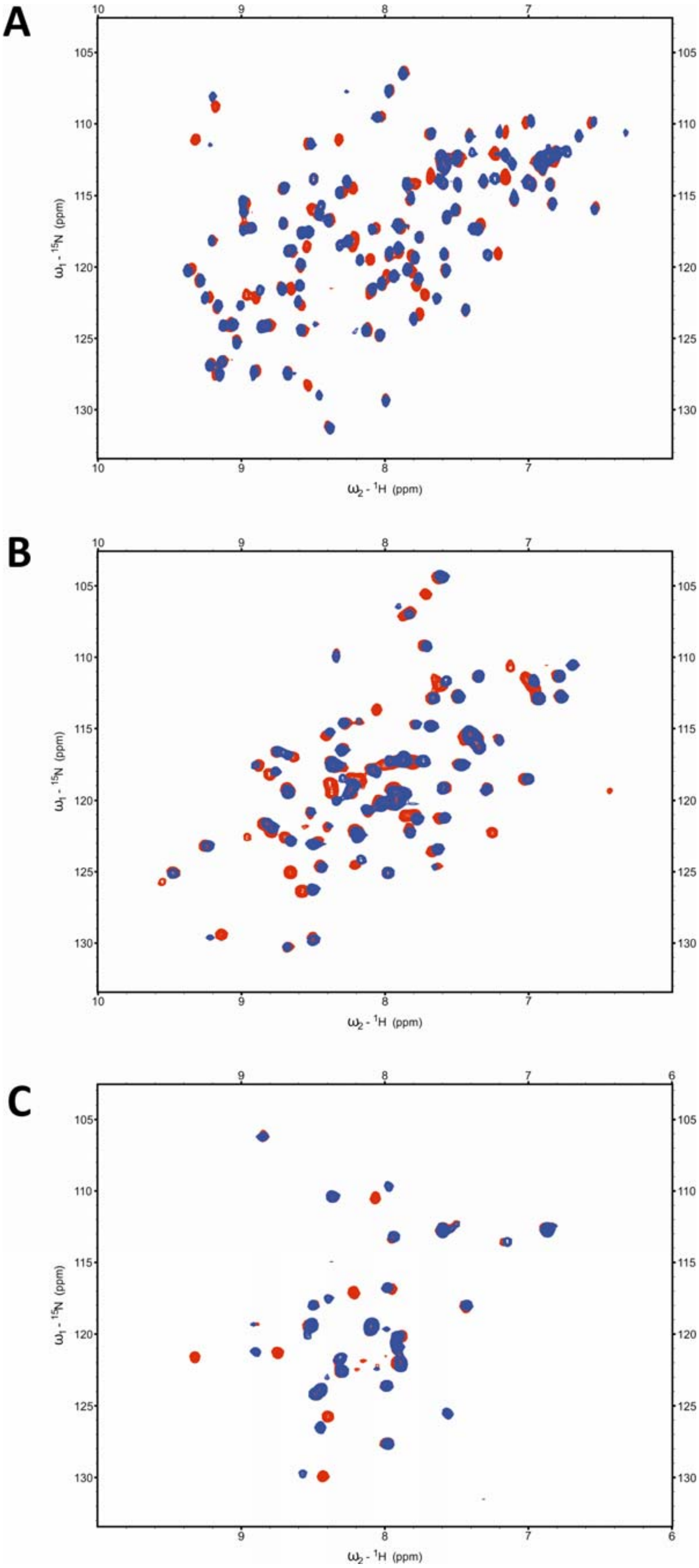

Table S1

RNA15 RRM

	PC1	PC2	PC3	PC4	PC5
**Position 1**	82.2	7.08	4.16	2.81	2.75
**Position 2**	84.63	6.42	3.34	2.78	2.84
**Position 3**	83.19	6.62	4.52	2.95	2.72
**Position 4**	85.52	6.11	3.05	2.62	2.69

TSTAR KH

	PC1	PC2	PC3	PC4	PC5
**Position 1**	75.73	8.63	6.06	4.82	4.69
**Position 2**	78.22	7.57	5.31	4.21	4.67
**Position 3**	76.35	8.38	6.13	4.73	4.4
**Position 4**	79.25	7.62	4.46	4.32	4.34

TUT4 CCHC-ZF3

	PC1	PC2	PC3	PC4	PC5
**Position 1**	58.2	12.97	9.51	9.67	9.65
**Position 2**	57.13	12.52	10.85	9.83	9.68
**Position 3**	57.78	12.86	9.87	9.75	9.74
